# Depicting Conformational Ensembles of α-Synuclein by Single Molecule Force Spectroscopy and Native Mass Spectroscopy

**DOI:** 10.3390/ijms20205181

**Published:** 2019-10-19

**Authors:** Roberta Corti, Claudia A. Marrano, Domenico Salerno, Stefania Brocca, Antonino Natalello, Carlo Santambrogio, Giuseppe Legname, Francesco Mantegazza, Rita Grandori, Valeria Cassina

**Affiliations:** 1 School of Medicine and Surgery, Nanomedicine Center NANOMIB, University of Milan-Bicocca, 20900 Monza, Italy; r.corti9@campus.unimib.it (R.C.); claudia.marrano@unimib.it (C.A.M.); domenico.salerno@unimib.it (D.S.); francesco.mantegazza@unimib.it (F.M.); 2 Department of Materials Science, University of Milan-Bicocca, 20125 Milan, Italy; 3 Department of Biotechnology and Biosciences, University of Milan-Bicocca, 20126 Milan, Italy; stefania.brocca@unimib.it (S.B.); antonino.natalello@unimib.it (A.N.); carlo.santambrogio@unimib.it (C.S.); 4 Scuola Internazionale Superiore di Studi Avanzati, SISSA, 34136 Trieste, Italy

**Keywords:** α-synuclein, single molecule force spectroscopy, intrinsically disordered proteins, native mass spectrometry

## Abstract

Description of heterogeneous molecular ensembles, such as intrinsically disordered proteins, represents a challenge in structural biology and an urgent question posed by biochemistry to interpret many physiologically important, regulatory mechanisms. Single-molecule techniques can provide a unique contribution to this field. This work applies single molecule force spectroscopy to probe conformational properties of α-synuclein in solution and its conformational changes induced by ligand binding. The goal is to compare data from such an approach with those obtained by native mass spectrometry. These two orthogonal, biophysical methods are found to deliver a complex picture, in which monomeric α-synuclein in solution spontaneously populates compact and partially compacted states, which are differently stabilized by binding to aggregation inhibitors, such as dopamine and epigallocatechin-3-gallate. Analyses by circular dichroism and Fourier-transform infrared spectroscopy show that these transitions do not involve formation of secondary structure. This comparative analysis provides support to structural interpretation of charge-state distributions obtained by native mass spectrometry and helps, in turn, defining the conformational components detected by single molecule force spectroscopy.

## 1. Introduction

Intrinsically disordered proteins (IDPs) play crucial regulatory roles in biological systems and lack a specific tertiary structure under physiological conditions [1,2,3,4]. Molecular characterization of IDPs requires description of the conformational ensembles populated by the disordered polymers in solution. Single-molecule approaches offer information on dynamic and heterogeneous ensembles, capturing distinct and less populated states, overcoming the limitations of average parameter assessment, intrinsic to bulk methods [5,6,7,8]. 

Usually employed in imaging mode [9,10], atomic force microscopy (AFM) can be used in single-molecule force spectroscopy (SMFS) to characterize the statistical distribution of distinct protein conformers in solution. Indeed, protein unfolding under the action of a pulling force has been demonstrated to characterize the molecular structure of tens of distinct proteins and to distinguish among different conformations induced by ligand binding or mutations [11,12,13]. In the case of the human, amyloidogenic IDP α-synuclein (AS), at least three major conformational states can be recognized [14,15,16]: random coil (RC), collapsed states stabilized by weak interactions (WI), and compact conformations stabilized by strong interactions (SI). The SMFS technique has been applied to explore the conformational space populated by the different structures of the protein, revealing distinct conformers of the molecular ensemble and structural effects of point mutations linked to familial Parkinson’s disease [4,14,15,16,17].

Pure AS in vitro, in the absence of interactors, is largely unstructured at neutral pH, with a small fraction of the population in collapsed states of different compactness, as revealed by NMR spectroscopy [18] and small angle X-ray scattering [19]. A particularly compact, globular state is populated in vivo, as indicated by in-cell NMR in neuronal and non-neuronal mammalian cell types [20]. Dopamine (DA) and epigallocatechin-3-gallate (EGCG) are known to bind AS and redirect the aggregation pathway toward soluble oligomers with different structure and toxicity [21,22].

Native mass spectrometry (native MS) has developed into a central tool for structural biology [23,24,25,26]. The analysis of charge states populated by globular and disordered proteins by native MS has shown effects of denaturants [27], stabilizers [28], metal binding [29], and protein–protein interactions [30], just to mention some examples. The application of native MS to free AS in solution reveals multimodal charge-state distributions (CSDs), which are suggestive of a conformational ensemble populated by different conformers, in line with the above-mentioned, in vitro and in vivo evidence [29,31,32,33]. The charge states obtained by proteins in electrospray have long been recognized as affected by protein compactness at the moment of transfer from solution to gas phase [27,34]. This effect can be rationalized by an influence of protein structure on solvent-accessible surface area [35,36,37] and apparent gas-phase basicity [38].

A large amount of evidence suggests that the ionization patterns of globular and disordered proteins are similarly affected by conformational properties [23,39,40,41]. Native MS has described conformational responses of AS to alcohols, pH, and copper binding consistent with NMR and other solution methods [29,33]. Native MS has also suggested that binding of DA and EGCG have distinct structural effects on AS soluble monomers [42,43]. While DA preferentially binds and stabilizes an intermediate form, EGCG promotes accumulation of the most compact AS conformer [42]. This different conformational selectivity could help rationalizing the different structure and toxicity of the resulting oligomers, although the two ligands have similar fibrillation-inhibition effects [42]. Nonetheless, the difficulty to capture IDP compact states by small-angle X-ray scattering and ensemble-optimization method has led to the hypothesis that IDP bimodal CSDs are artifacts resulting from a bifurcated ESI mechanism, rather than distinct components reflecting structural heterogeneity of the original protein sample [44]. The aim of this work is to describe AS conformational ensemble and its response to ligands by orthogonal and highly sensitive biophysical techniques, such as SMFS, in order to test the effect of ligand binding in solution and help interpretation of the available native-MS data on AS and IDPs in general. It is found that, while spectroscopic methods sensitive to secondary structure do not capture these conformational transitions, SMFS and native MS reveal rearrangements of the conformational ensembles, consistent with a loss of structural disorder induced by the ligands.

## 2. Results

### 2.1. Single Molecule Force Spectroscopy (SMSF)

**Figure 1:**
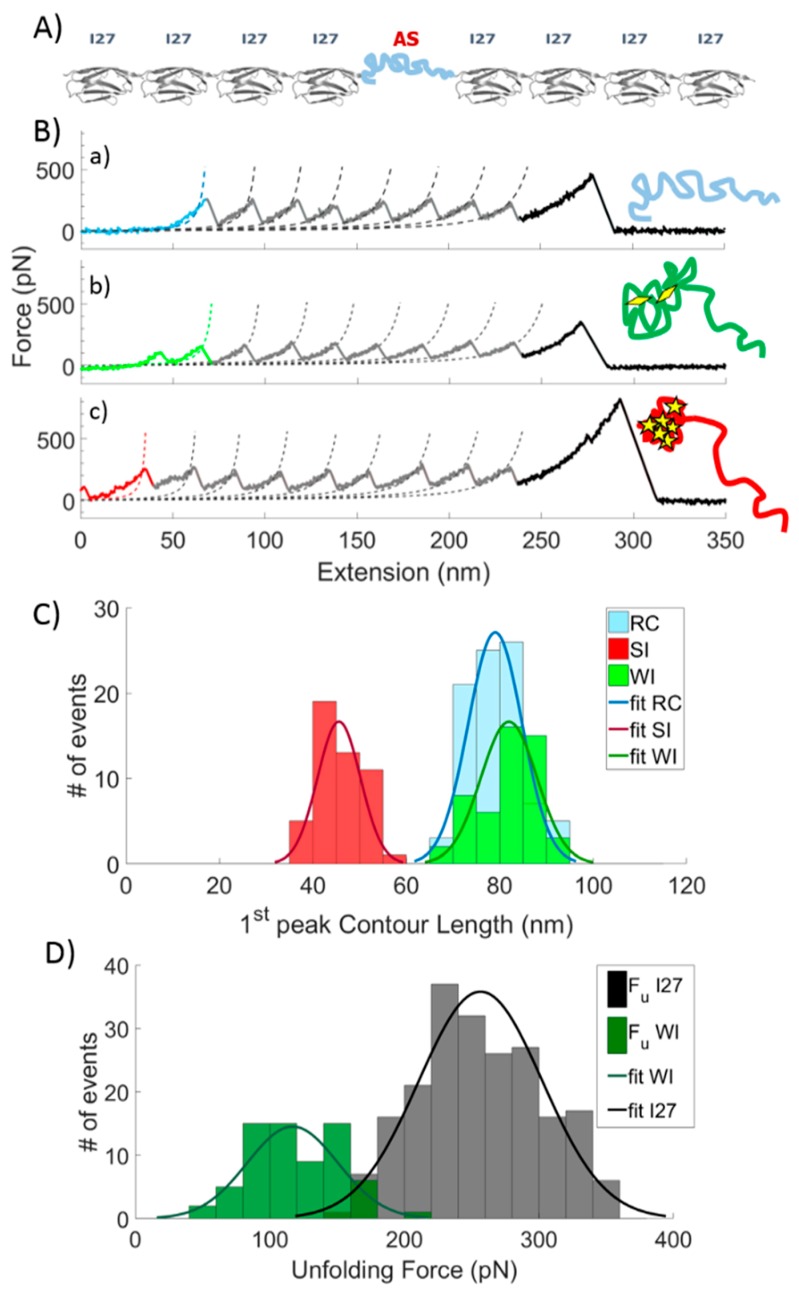
Representative single molecule force spectroscopy (SMFS) recording of α-synuclein (AS) polyprotein and relative statistical analysis. (**A**) Polyprotein construct encompassing the AS full-length polypeptide chain for SMFS experiments. (**B**) Representative force curves of the mechanical unfolding of the polyprotein in distinct conformations stabilized by RC (a), WI (b), and SI (c). Dotted lines are worm-like-chain (WLC) fits to the force-extension curves with free contour length L_C_ and a fixed persistence length L_p_ = 0.36 nm (see Figure S2 for raw data). Sketches of AS conformations are shown on the right. Diamonds represent weak interactions stabilizing the AS protein, while stars represent strong interactions. (**C**) Statistical distribution of the contour length of the first peak for RC (L_C_ = 79 ± 6 nm), WI (L_C_ = 82 ± 6 nm), and SI (L_C_ = 46 ± 5 nm) conformations. Solid lines represent the Gaussian fits of the histograms. (**D**) Unfolding force statistical distribution of WI (F_WI_ = 117 ± 34 pN) and I27 modules (F_I27_ = 257 ± 46 pN).

The SMFS experiments have been performed on a polyprotein construct containing eight repeats of titin immunoglobulin-like domain (I27) and one grafted AS domain [45,46,47]. A schematic representation of the polyprotein construct and typical unfolding curves in the absence of ligands are reported in Figure 1A,B.

As apparent from Figure 1B, the observed SMFS curves show the typical “sawtooth” pattern, in which the initial part is related to the presence of AS and it is characterized by different mechanical resistances to the unfolding. Each following regular peak is due to the unfolding of an individual I27 domain. Every curve was fitted by means of the worm-like-chain (WLC) model to extract the contour length L_C_ of each peak (both for I27 and AS) [48]. Consistent with the presence of a heterogeneous conformational ensemble, three distinct patterns can be recognized by analyzing the L_C_ of the first peak (Figure 1B,C). A first class of curves displays L_C_ = 79 ± 6 nm (light blue curve, first line of Figure 1B); a second class is characterized by L_C_ = 82 ± 6 nm and by the presence of at least one small peak before the first regular peak (green curve, second line of Figure 1B); a third class displays L_C_ = 46 ± 5 nm and it is characterized by the presence of an additional peak whose height is comparable to the one related to an I27 unfolding event (red curve, third line in Figure 1B). In detail, the light blue curves are ascribed to unstructured conformations of AS and classified as random coil (RC), since no additional peak is detected in the first ~80 nm. The green curves display small (one or more) peaks corresponding to an unfolding force (F_WI_ = 117 ± 34 pN) sensibly lower than I27 (F_I27_ = 257 ± 46 pN, Figure 1D, Figure S1 and Table S1). These curves are interpreted as representative of a collapsed state of AS mainly stabilized by weak interactions (WI), characterized by an energy barrier to overcome smaller than the one involved in the I27 unfolding. The third and the latter type of curves, characterized by a shorter L_C_ is assigned to a collapsed state of AS, mainly stabilized by strong interactions (SI), which presents resistance to unfolding similar to the one shown by the highly mechanostable protein I27. The extension of the first peak (in the curves assigned to the RC conformation) and that of the first of the higher peaks (in the curves assigned to the WI) are all around 80 nm. These peaks occur when AS is completely extended and flanked by eight I27 folded modules. The measured length is due to the contribution of the eight folded I27 modules (a folded module of I27 is 3 nm long, i.e., 3 nm × 8 = 24 nm), the length of eight linkers between each protein module (a linker is 2 aa, i.e., 8 × 0.36 nm × 2 = 5.76 nm) [47], and the length of the completely extended AS (i.e., 140 aa × 0.36 nm = 50.4 nm). By summing all the contributions, one obtains a total extension of 80.16 nm, which is coherent with the measured values. The subset of curves presenting a L_C_ of the first peak higher than 95 nm, which could be associated with an undesired misfolding event of a I27 module [49], was discarded.

### 2.2. Effect of DA and EGCG on the Conformational Ensemble

**Figure 2:**
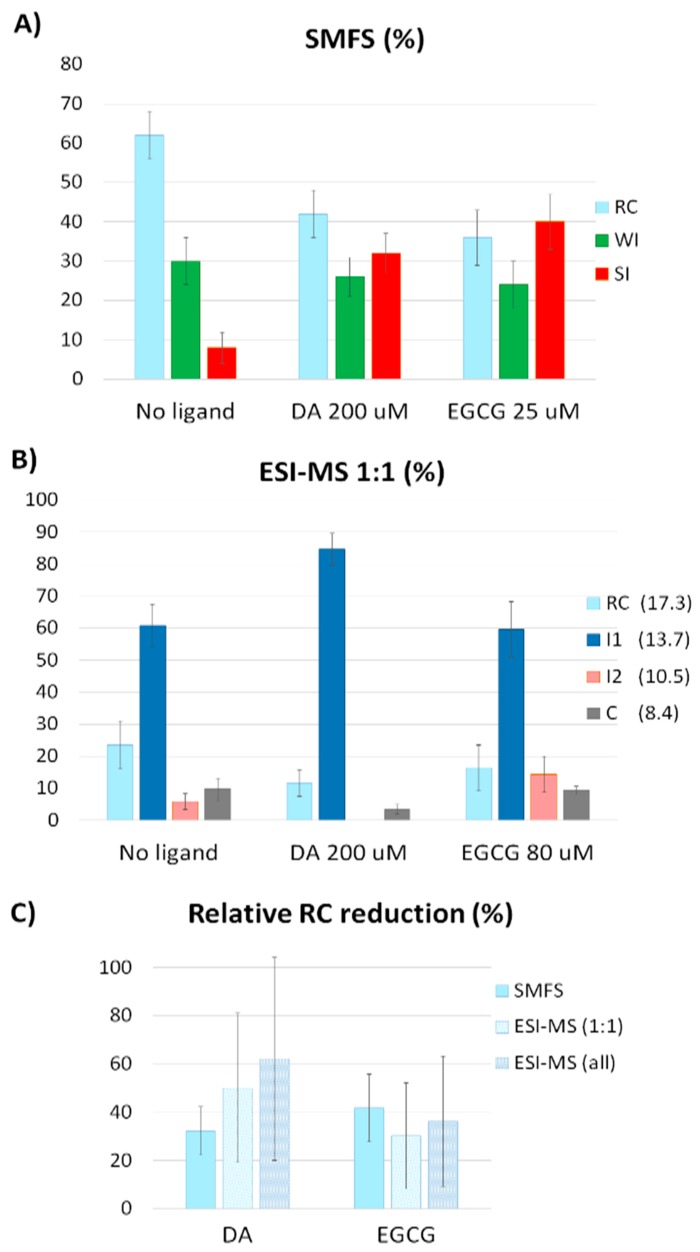
Species distributions as obtained by (**A**) SMFS and (**B**) native MS. The intensity-weighted average charge of the peak envelopes is reported in brackets (i.e., RC = 17.3; I1 = 13.7; I2 = 10.5; C = 8.4). Error bars in panel (A) represent the standard deviation calculated for the normal distribution. Error bars in panel (B) represent the standard deviations from three independent experiments. (**C**) RC reduction in response to ligand binding, relative to the free protein, as obtained by SMFS and native MS, considering the 1:1 protein:ligand complexes (1:1) or the cumulative MS data (all). Error bars in panel (C) represent the propagated standard deviation.

The SMFS measurements were repeated in the presence of either 200 μM DA or 25 μM EGCG (see Figures S3–S6 and Tables S2–S6 for more details). These concentrations were chosen to compare SMFS results with native-MS data [42]. The statistical distributions of AS conformations obtained by SMFS in the presence or absence of ligands are reported in Figure 2A. In solution, at neutral pH and without ligands, AS behaves partially as RC (62% of the molecules) and partially populates collapsed states (~30%, mainly stabilized by WI and ~8%, mainly stabilized by SI), consistent with previously reported SMFS data [14,15,16]. The addition of either ligand leads to a loss of the RC conformation in favor of the SI conformation, with the most pronounced effect of EGCG (drop of RC from 62% to 36%). The same conditions had been investigated by native MS, showing the presence of intermediate states (I1 and I2), together with random coil (RC) and compact (C) conformations (Figure 2B). 

A quantitative comparison between the species distributions obtained by SMFS and native-MS data is shown in Figure 2C. An intrinsic difference between SMFS and MS concerns the discrimination between free and ligand-bound protein molecules, which is possible only by the latter technique. Thus, native-MS data in Figure 2C have been processed by two alternative ways. In one case, only signals of the 1:1 protein:ligand complexes have been considered. This procedure yields more reliable information on the conformational changes induced by ligand binding but is, at the same time, not exactly comparable to the blind molecular selection performed by SMFS. Thus, “cumulative” MS data are also shown (labeled as ESI-MS(all) in Figure 2C), derived by Gaussian fitting of the artificial CSD obtained by the summation of the species-specific CSDs corresponding to the different binding stoichiometries, including the free protein. In either way, the aggregated data for the unstructured (RC) component, represented as relative change from the reference condition of the protein in the absence of ligands, indicate a remarkable loss of the most disordered conformation induced by ligand binding, as assessed by both techniques.

### 2.3. Comparison to CD and FTIR

**Figure 3:**
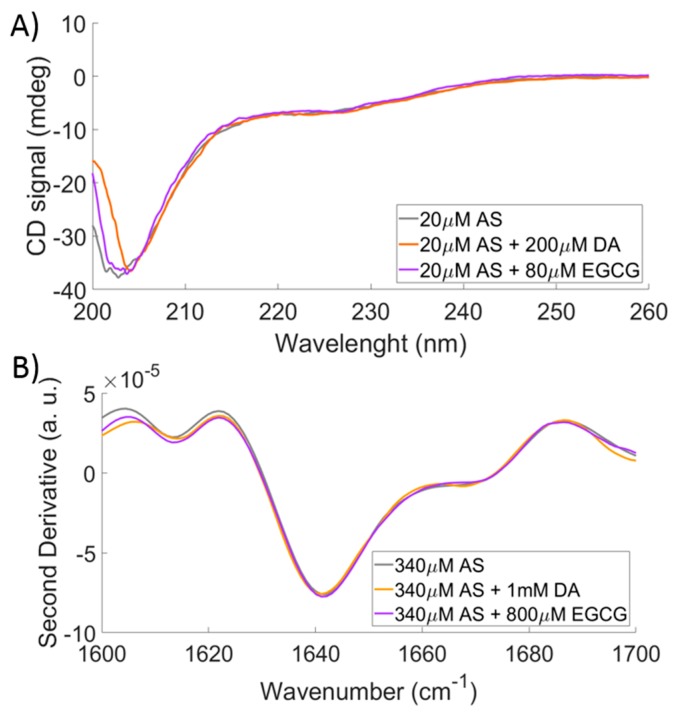
Secondary-structure content as obtained by CD and Fourier-transform infrared spectroscopy (FTIR) techniques. (**A**) Far-UV CD spectra of 20 μM AS in PBS buffer in the absence of ligands (gray), in the presence of 200 μM DA (orange) or 80 μM epigallocatechin-3-gallate (EGCG) (purple). (**B**) Second derivatives in the Amide I region of the FTIR absorption spectra of 340 μM AS in deuterated PBS buffer in the absence of ligands (gray) and in the presence of 1 mM DA (orange) or 800 μM EGCG (purple).

For comparison with complementary spectroscopic methods, sensitive to protein secondary structure, far-UV circular dichroism (CD) and Fourier-transform infrared spectroscopy (FTIR) analyses were performed. Representative results are reported in Figure 3. It can be noted that AS spectra in the presence or absence of the ligands, acquired by either technique under the same conditions employed for SMFS experiments, are almost superimposable. Thus, bulk methods probing secondary structure do not capture the conformational changes induced by ligand binding in monomeric AS in solution.

## 3. Discussion

The results reported here provide direct evidence of the different conformers populated by AS in solution and the structural effects elicited by ligand binding, resulting in a rearrangement of the conformational ensemble [50]. The structural heterogeneity of free AS in solution captured by SMFS is consistent with previous reports by the same approach [14,15], as well as with results from native MS [23,29,33,42], computational simulations [51,52], and chemical crosslinking [53], indicating the presence of at least three different conformational states characterized by different degrees of intramolecular interactions. Furthermore, SMFS is applied here for the first time to probe the effects of the fibrillation inhibitors DA and EGCG on AS conformational properties in solution. 

Since the reliability of CSD analysis in the investigation of IDP conformational ensembles by native MS has been questioned [44], the SMFS results obtained in this work are compared to native-MS data. In analogy with SMFS, the CSD analysis of nano-ESI-MS spectra identifies, in addition to the RC component, the presence of three non-RC components, namely the intermediate species I1, I2, and the compact conformation C. Furthermore, both techniques indicate a loss of the most disordered component in response to ligand binding, resulting in the accumulation of the more structured species. Thus, not only the presence of multimodal profiles is confirmed by both techniques, but also a reorganization of the conformational ensemble in the same direction is consistently indicated in the presence of ligands. 

Nonetheless, the structural intermediates detected by SMFS and native MS cannot be related in a straightforward way. These discrepancies can be due to the fact that the physical properties detected by the two techniques are different. While SMFS discriminates protein structures according to their mechanical stability under an external tension (quantified by the unfolding force), native MS is affected by structural compactness (quantified by the acquired net charge). Different compaction levels can correspond to similar unfolding force and vice-versa. Accordingly, the WI state, as detected by SMFS, is characterized by a number of variable peaks ranging from 1 to 3 different species, which could be compatible with different AS compaction states. Furthermore, the SMFS instrumental noise, related to the minimum measurable force (around 20pN) limits the minimal detectable unfolding force, below which the less stable AS compact states are counted as RC molecules. 

It should also be noted that the conformations with lower unfolding force, as detected by SMFS, could include some components with higher charge-state detected by ESI-MS. This hypothesis can be verified by comparing the two techniques in terms of the response of the RC component to the binding of the ligand. Indeed, upon binding of either ligand there is a compatible trend of loss in such a component, as observed by both techniques, in favor of more compact structures (native MS) or stronger interactions (SMFS). Therefore, interpreting the low-charge components of CSDs as collapsed and partially structured conformational states leads to compatible pictures delivered by SMFS and native MS. Both techniques reveal the presence of partially structured conformers, thus suggesting that the bimodal or multimodal CSDs detected by native MS do not simply reflect artefacts of the ESI mechanism. It is worth pointing out that the conditions employed in this work do not lead neither to AS oxidation (Figure S7) nor to AS oligomerization, which requires incubation at 37 °C, shaking and higher protein concentrations [54], as also indicated by the lack of higher-order aggregates in native-MS spectra [42].

It cannot be ruled out that different ionization and/or transmission efficiency of compact and extended protein ions in native MS might lead to distortions of the apparent molecular ensemble, adding to the difficulties of direct comparison with SMFS data. Indeed, it has been suggested that folded and unfolded molecules could undergo different ESI mechanisms, resulting in different signal yields [55]. However, this effect seems to be protein-specific, since quantitative agreement with solution methods has been observed describing, for instance, the pH-dependent unfolding transition of cytochrome *c* [56]. The underlying mechanism has been identified in the different hydropathy of the exposed regions of normally folded proteins in different conformational states, which could affect their surface activity inside ESI droplets [55]. Such an effect is expected to be much more modest for IDPs, which lack a structured hydrophobic core and whose collapsed conformations are mostly promoted by electrostatic interactions [57]. More systematic, quantitative comparison between native MS and solution methods will be required to further elucidate this point. This first comparative study between a single-molecule technique and CSD analysis by native MS supports the feasibility of combined approaches to describe IDP molecular ensembles.

Based on this study, it seems safe to conclude that SMFS and structural interpretation of CSDs consistently indicate the simultaneous presence of collapsed and partially structured conformers of AS monomer in solution and, most importantly, reveal induced-folding transitions elicited by ligand binding. Furthermore, this study shows that single-molecule protein unfolding can capture changes in AS conformational landscape, induced by variable solution conditions, with remarkable sensitivity and reproducibility. These results indicate that the conformational ensemble depicted by two orthogonal biophysical principles is heterogeneous and reshaped in the same direction by ligand binding. 

Another implication of this study is that the AS conformational transitions detected by SMFS under these conditions should not be interpreted in terms of secondary structure formation [14,15]. Indeed, the measured WI and SI components cannot be simply seen as the distinct contributions of van-der-Waals interactions and ordered secondary structure, respectively. In fact, an increase of almost 30% in the SI component, as observed here, would be detected by CD and FTIR spectroscopies, if ascribable to secondary structure. It is conceivable that the AS conformational components detected by SMFS under these conditions differ by contact order, type, and number of interactions, within a picture of similar secondary-structure content. Hence, a new structural interpretation of SMFS data is proposed, in particular for the SI population, differing from the one reported in the literature [14,15], where the SI component was directly associated with the presence of secondary structure.

This comparison points out that the ion-sorting mechanism inherent to MS analyses makes the MS methods more comparable to single-molecules approaches, rather than to bulk spectroscopic techniques, and underscores the importance of multi-technological approach to ensemble characterization. Nevertheless, the WI population detected by SMFS and the intermediate species (I1 and I2) detected by native MS do not necessarily coincide. Actually, two intermediates are detected by MS and only one by SMFS and the WI species found by SMFS does not respond to ligands, while the MS-detected intermediates do. These results indicate that both techniques capture the decrease in structural disorder induced by the ligands, but they describe the partially structured species of the conformational ensemble in different ways. In particular, it seems that the collapsed and partially structured species detected by MS contribute cumulatively to the SI component by SMFS, while the WI component by SMFS does not find correspondence in the MS spectra. These interactions could be too weak to survive the ionization/desolvation step.

## 4. Materials and Methods 

### 4.1. Cloning, Expression, and Purification of the (I27)4_AS_(I27)4 Polyprotein

In order to obtain a (I27)4_AS_(I27)4 polyprotein, consisting of a single AS molecule, flanked by four repetitions of titin immunoglobulin-like domain (I27) at the N-terminus and at the C-terminus, the cDNA of the human *AS* (NP_000336) was cloned in the pRSet.A(I27)8 expression vector [47], taking advantage of the NheI restriction site placed in the middle of (I27)8 encoding sequence. A mutagenic PCR was performed on the pEGFP_AS vector [58] to delete the start and stop codons and to insert a NheI restriction site at both extremities of the *AS* gene. The PCR was carried out using the Q5® High-Fidelity DNA Polymerase (NEB, cat. #M0491) with the following primers: forward primer 5’ AAAAGCTAGCGATGTATTCATGAAAGGAC 3’, reverse primer 5’ AATTGCTAGCGGCTTCAGGTTCGTAG 3’, (in bold, the NheI restriction site). After sequencing, the pRSet.A (I27)4_AS_(I27)4 vector was used to transform BL21(DE3) *Escherichia coli* cells. Transformed cells were grown in Luria-Bertani medium at 37 °C until they reached an OD600 of 0.4–0.6 and the expression of the polyprotein was induced overnight at 22 °C by the addition of 1 mM IPTG. Cells were subsequently harvested by centrifugation and resuspended in lysis buffer (50 mM Na_2_HPO_4_, 300 mM NaCl, 10 mM imidazole, 4% Triton™ X-100, and 0.5 mM phenylmethylsulfonyl fluoride) before sonication on ice. The purification was performed by gravity flow column ion metal affinity chromatography (IMAC), taking advantage of the 6× His-tag present at the N-terminus of the polyprotein. The soluble fraction of cell lysate was incubated on Ni+-NTA resin (Roche, cat. #05893682001) for 1 h at 4 °C with gentle agitation. The washing step was carried out in 50 mM Na_2_HPO_4_, 300 mM NaCl added with 20 mM imidazole, elution was achieved in the same buffer, added with 250 mM imidazole. The presence of the protein in the eluted fractions was verified by SDS-PAGE on a 4–12% polyacrylamide gel (InvitrogenTM, ThermoFisher Scientific, cat. #NW04120BOX) stained with Coomassie Brilliant Blue.

### 4.2. AFM—Single Molecule Force Spectroscopy

SMFS experiments were carried out on a Nanowizard II (JPK Instruments, Berlin, Germany) at room temperature. Prior to each experiment, every cantilever (Si_3_N_4_, Bruker MLCT-BIO, Cantilever D, Nominal spring constant k = 0.03 N/m) was individually calibrated using the Equipartition Theorem in the JPK software. Approximately 20 μL of protein (at a concentration of ~2 µM) were deposited onto an evaporated gold coverslip and allowed to adsorb for about 15 min. After this time, 1.8 mL of PBS buffer (pH 7.4, 150 mM) were added to reach an overall protein final concentration of ~20 nM. Constant-velocity, single-molecule pulling experiments were performed at 1 μm/s, with a recorded rate of 4096 Hz. Each experiment was carried out in fresh PBS buffer, to which EGCG (stock diluted in PBS) and DA (stock diluted in acidic MilliQ, pH 4) (stored at 4 °C protected from light) were added to reach the desired final concentration. Each solution was filtered on a filter screen with a porosity of 0.2 μm before each experiment. 

### 4.3. AFM Data Analysis

The resulting force curves were then processed by means of both the JPK-Data Processed software (JPK Instruments, Berlin, Germany) and MATLAB custom-written software. The contour length (L_C_) of each peak (both I27 and AS) was calculated by means of WLC fit as a single parameter, while the persistence length (L_P_) was kept constant (0.36 nm) [59]. Only curves with a single clear detachment peak, at least seven I27 peaks, and traces with a spurious signal below 45pN in the first 25 nm of the force-extension were considered.

### 4.4. Native-MS Experiments

Nano-ESI-MS data were taken from Konijnenberg [42]. In particular, nano-ESI-MS spectra were collected after 10-minute incubation of protein-ligand mixtures in 10 mM ammonium acetate, pH 7.4, at a final AS concentration of 20 μM. Quantification from native-MS data was based on Gaussian fitting of CSDs, upon transformation to x = z abscissa axis. The reported values refer to the area of the components obtained for the protein in the absence of ligand and for the 1:1 AS:ligand complexes, from three independent experiments.

### 4.5. CD and FTIR Experiments

CD and FTIR analyses were performed as previously described [43]. In particular, Far-UV CD spectra of 20 μM AS in PBS buffer were acquired on a J-815 spectropolarimeter (JASCO Corp., Tokyo, Japan) under the following instrumental settings: data pitch, 0.1 nm; scan speed, 20 nm/min; bandwidth, 1 nm; accumulation spectra, 2. A 1-mm path length quartz cuvette was employed. FTIR spectra of 340 μM AS in deuterated PBS buffer were acquired on a Varian 670-IR spectrometer (Varian Australia Pty. Ltd., Mulgrave, VIC, Australia) under the following instrumental settings: resolution, 2 cm^−1^; scan speed, 25 kHz; scan coadditions, 1000; apodization, triangular; nitrogen-cooled mercury cadmium telluride detector. A temperature-controlled transmission cell with two BaF_2_ windows separated by a 100-μm Teflon spacer was employed. Representative spectra from three independent experiments are shown.

## 5. Conclusions

Single-molecule description of AS conformational ensemble in solution detects differently structured components that are overseen by bulk spectroscopic methods, which probe secondary structure, but are consistent with the different degrees of compactness suggested by CSD analysis. Thus, although ion-mobility studies and molecular-dynamics simulations have shown that IDPs rearrange in the gas phase in a charge-dependent fashion [40], the extent of ionization at the moment of transfer from solution to gas phase, i.e., CSDs, seems to reflect structural heterogeneity in solution rather than ESI artifacts. This correspondence is experimentally established here, independently of assumptions on the underlying ESI mechanism. Combined description by orthogonal biophysical methods can provide valuable constraints for computational simulations of IDP conformational ensembles in the presence or absence of interactors [51]. 

## Supplementary Materials

Supplementary materials can be found at https://www.mdpi.com/1422-0067/20/20/5181/s1.

## Abbreviations

AS	α-synuclein
C	compact structure detected in native MS
CD	circular dichroism
CSDs	charge state distributions
DA	dopamine
EGCG	epigallocatechin-3-gallate
ESI-MS	electrospray ionization mass spectrometry
F	unfolding force
FTIR	Fourier-transform infrared spectroscopy
I1, I2	Intermediate 1 and 2 detected in native MS
I27	27th titin immunoglobulin-like domain
IDP	intrinsically disordered protein
IDPs	intrinsically disordered proteins
L_C_	contour length
L_P_	persistence length
native MS	native mass spectrometry
NMR	nuclear magnetic resonance
RC	random coil
SAXS-EOM	small-angle X-ray scattering and ensemble-optimization method
SI	strong interactions
SMFS	single molecule force spectroscopy
WI	weak interactions
WLC	worm-like-chain
