# Supplementary material for: Depicting Conformational Ensembles of α-Synuclein by Single Molecule Force Spectroscopy and Native Mass Spectroscopy
Source: Int J Mol Sci. 2019 Oct 19;20(20):5181. doi: 10.3390/ijms20205181 (PMC6829300; doi:10.3390/ijms20205181)
Supplement: Supplementary file 1 [file ijms-20-05181-s001.pdf]

Article on Special Issue: Functionally Relevant Macromolecular Interactions of Disordered Proteins 2019

Depicting Conformational Ensembles of α-Synuclein by Single Molecule Force Spectroscopy and Native Mass Spectroscopy.

Supporting Information

Roberta Corti [1,2], Claudia A. Marrano [1], Domenico Salerno [1], Stefania Brocca [3], Antonino Natalello [3], Carlo Santambrogio [3], Giuseppe Legname [4], Francesco Mantegazza [1], Rita Grandori [3,*], Valeria Cassina [1*]

[1] School of Medicine and Surgery, Nanomedicine Center NANOMIB, University of Milan-Bicocca, 20900, Monza, Italy; claudia.marrano@unimib.it (C.A.M.); domenico.salerno@unimib.it (D.S.); francesco.mantegazza@unimib.it (F.M.)

[2] Department of Materials Science, University of Milan-Bicocca, 20125, Milan, Italy; r.corti9@campus.unimib.it (R.C.)

[3] Department of Biotechnology and Biosciences, University of Milan-Bicocca, 20126, Milan, Italy; stefania.brocca@unimib.it (S.B.); antonino.natalello@unimib.it (A.N.); carlo.santambrogio@unimib.it (C.S.);

[4] Scuola Internazionale Superiore di Studi Avanzati, SISSA, 34136, Trieste, Italy; giuseppe.legname@sissa.it (G.L.)

* Correspondence: valeria.cassina@unimib.it (V.C.); rita.grandori@unimib.it (R.G.)

Characterization of the I27 module: contour length and unfolding force

As a consequence of the described unfolding procedure, the several I27 modules of the polyprotein are unfolded under the mechanical action of the applied pulling force. As a result, a series of peaks in the force-extension curve are detected. Each peak is analyzed considering its force maximum value (F_U), which corresponds to the force necessary to unfold a single I27 module. Furthermore, the force-extension data can be fitted to the Worm-Like-Chain (WLC) model in order to extract the asymptotic value (contour length, L_C) for the extension of each I27 module, when the protein chain is completely extended.

Figure S1 and Table S1 illustrate the statistical distribution of the F_U and L_C values for I27 as obtained in the absence or presence of ligands. As shown by the reported data, there is no appreciable change in the F_U and L_C behavior of I27 due to the addition of DA or EGCG. Thus, the statistical values of F_U and L_C are collectively evaluated for all the I27 module (F_U of I27 in Fig.1D).

	**No Ligands**	**200 µM DA**	**25 µM EGCG**	**Total**
**F_U - I27 (pN)**	274 ± 42	227 ± 40	271 ± 39	257 ± 46 (*)
**L_C - I27 (nm)**	28.2 ± 1.1	28.0 ± 0.7	28.1 ± 0.9	28.1 ± 0.9

Table S1: *Measured average values +/- standard deviation of the unfolding force F_U and contour length L_C measured for the I27 module for each force-extension curve. The last column reports the cumulative statistical values from data acquired under the different tested conditions (no ligand, DA, EGCG) (* Fig.1D of the main paper).*

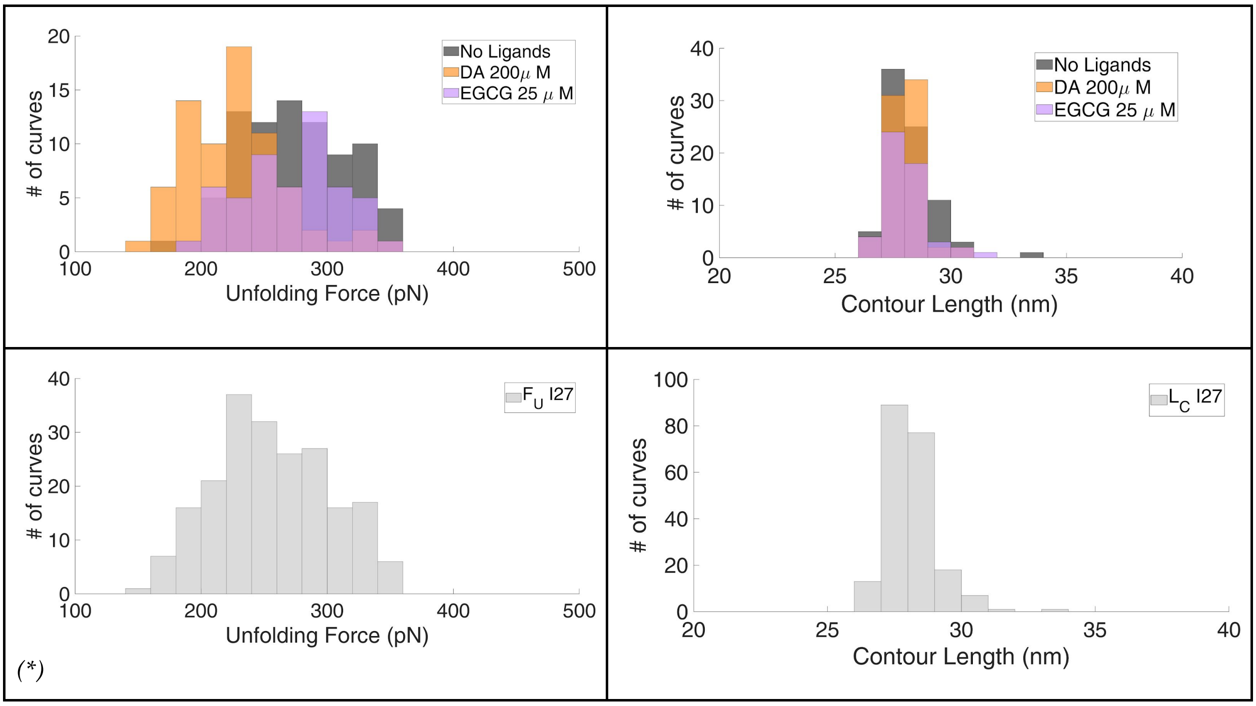

Figure S1: *Statistical distribution of the unfolding force F_U (left column) and of the contour length L_C (right column) averaged over all the I27 modules, for each force-extension curve. The upper panels represent results as a function of the different tested conditions (no ligand, DA, EGCG), as indicated by the color legend. The lower panels show the cumulative statistical distribution of the data. (* Fig.1D of the main paper)*

Force-extension representative curves

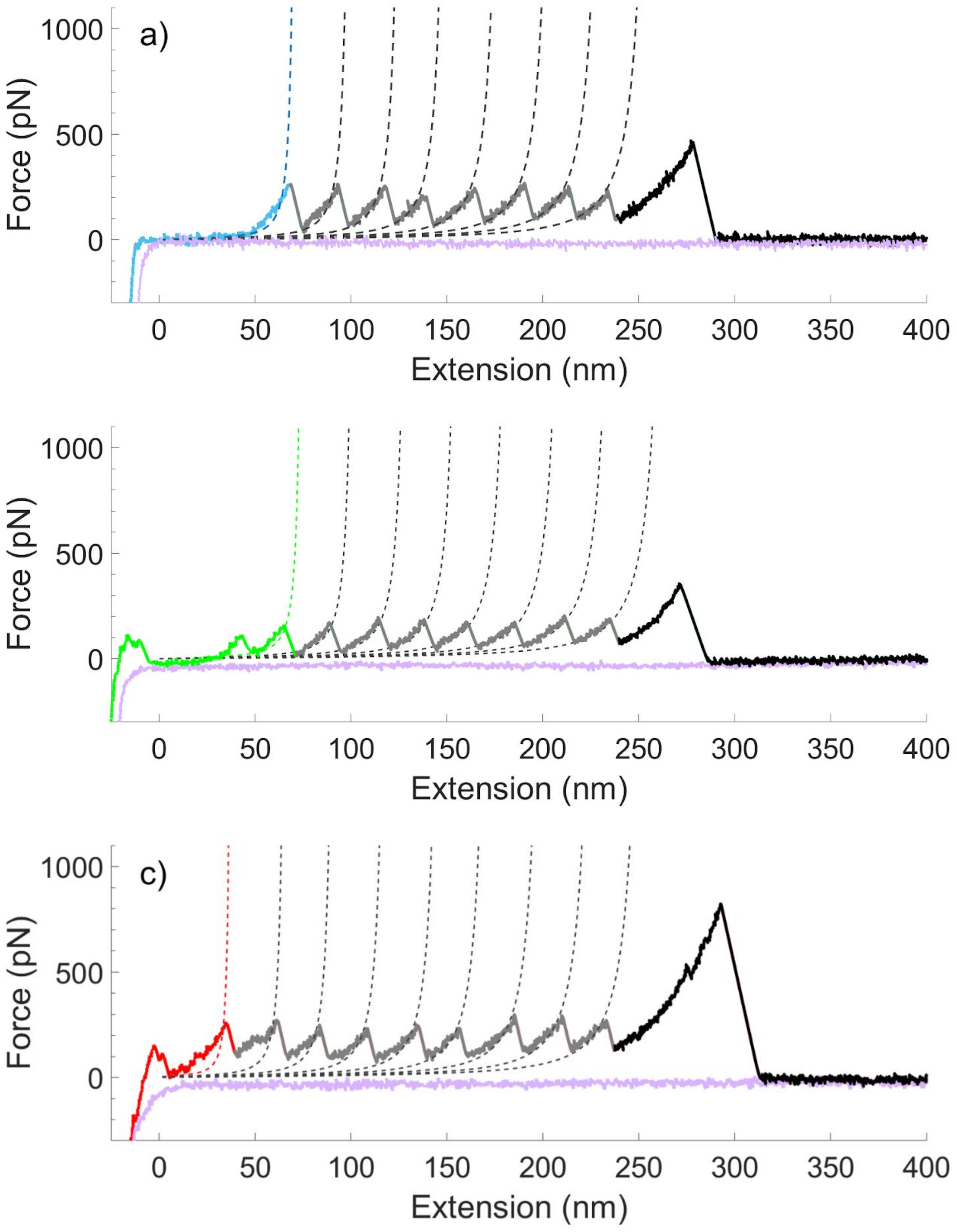

Figure S2: *Representative force curves for mechanical unfolding of the polyprotein in the conformations respectively classified as RC (a), WI (b), and SI (c). Dotted lines are WLC fits to the force-extension curves. The approaching curves are plotted in violet. The WLC fit shows poor agreement in the 1st and 2nd peak (especially in panel (c)) due to the presence of unfolding intermediates in the I27 modules [59].*

First peak in the force-extension curves: contour length and unfolding force

Table S2 shows the contour length values (mean +/- std) of the first peak of each curve for the three conformations in the presence or absence of ligands. The last column ("Total") reports the cumulative LC values obtained in different conditions and reported in the paper (see Fig.1C).

	1st peak contour length L_C (nm)			
	**No Ligands**	200 µM DA	25 µM EGCG	**Total (**)**
**Random Coil**	79.1 ± 5.7	78.7 ± 5.2	79.5 ± 7.0	79.0 ± 5.8
**Strong Interactions**	40.7 ± 1.2	47.0 ± 3.9	45.1 ± 5.0	45.6 ± 4.6
**Weak Interactions**	82.0 ± 5.8	81.9 ± 5.8	82.1 ± 6.9	82.0 ± 6.0

Table S2: *Measured average values +/- standard deviation of the contour length of the first peak for each force-extension curve. The last column reports the cumulative statistical values of the data acquired under the different tested conditions (no ligand, DA, EGCG) (** Fig.1C of the main paper).*

Table S3 shows the values of the unfolding force (mean +/- std) measured in correspondence of the first peak for the three conformations (RC, SI and WI). The last column ("Total") reports the cumulative F_U statistical values, regardless of the different buffer conditions (no ligand, DA, EGCG). The results indicate that ligands do not affect the contour length and the unfolding force of the first peak, although they induce changes in the statistical distributions of conformations.

	1st peak unfolding force F_U (pN)			
	**No Ligands**	200 µM DA	25 µM EGCG	**Total**
**Random Coil**	221 ± 34	187 ± 37	215 ± 45	207 ± 40
**Strong Interactions**	208 ± 26	195 ± 50	217 ± 32	206 ± 42
**Weak Interactions**	218 ± 40	196 ± 36	234 ± 33	214 ± 39

Table S3. *Measured average values +/- standard deviation of the unfolding force of the first peak for each force-extension curve. The last column reports the cumulative statistical values for data acquired under the different tested conditions (no ligand, DA, EGCG).*

Fig.S3 shows the histograms of the unfolding force (left column) and the contour length (right column) of the first peak. These data indicate that the unfolding force value and the contour length of the first peak are not affected by the presence of the ligands.

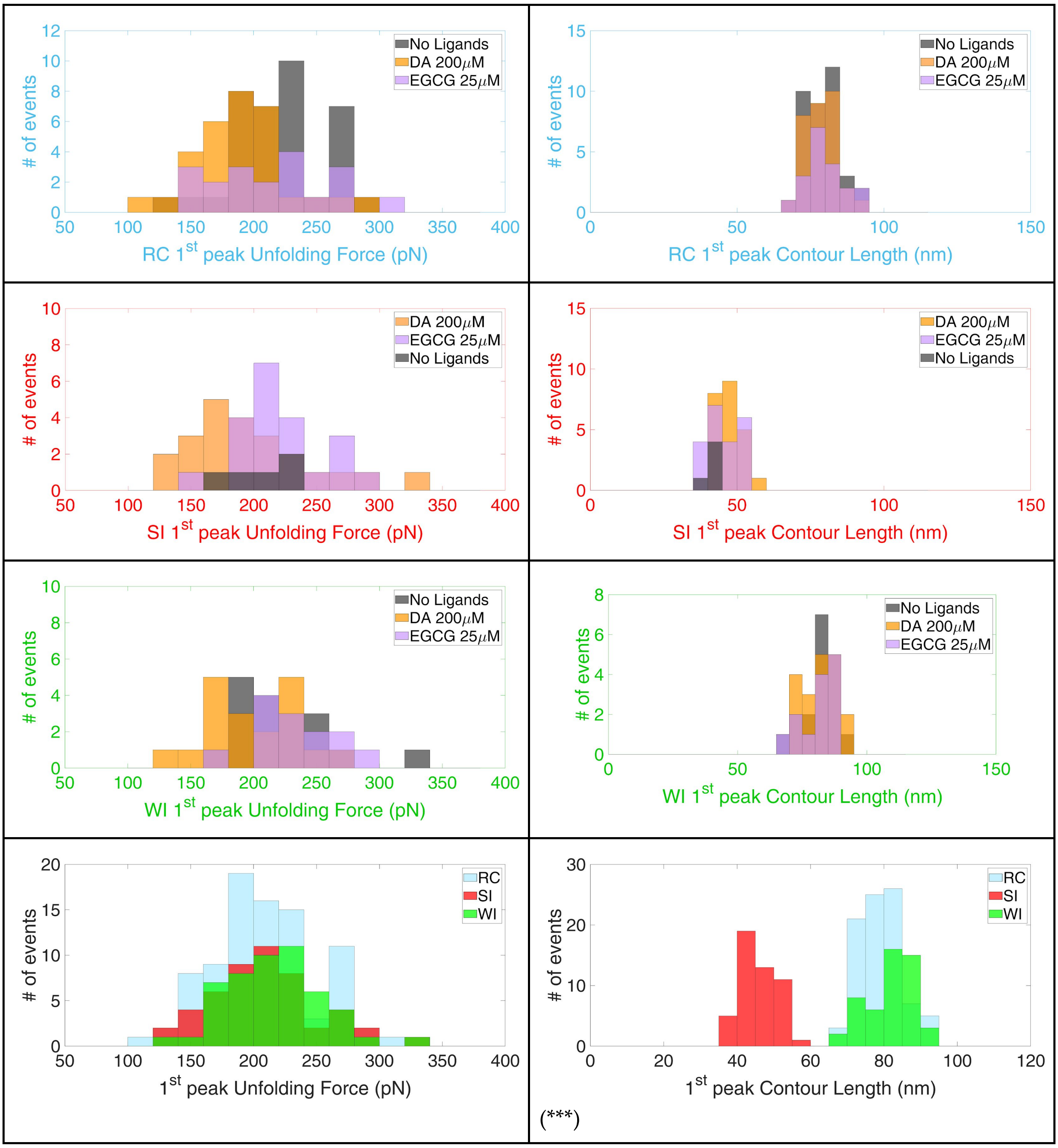

<u>Figure S3</u>: *Statistical distribution of the unfolding force F_U (left column) and contour length L_C (right column) for the first peak of each force-extension curve. Data are shown for the three main conformations (RC, SI, WI) as a function of the different tested conditions (no ligand, DA, EGCG), as indicated by the color legend. The lower panels show the cumulative statistical distribution of the same data. (*** Fig.1C of the main paper)*

Characterization of the Mechanically Weak Interaction peaks

Fig.S4 shows the distribution of the unfolding force of the mechanically weak interactions peaks compared with the corresponding first higher peak of the same force-extension curve.

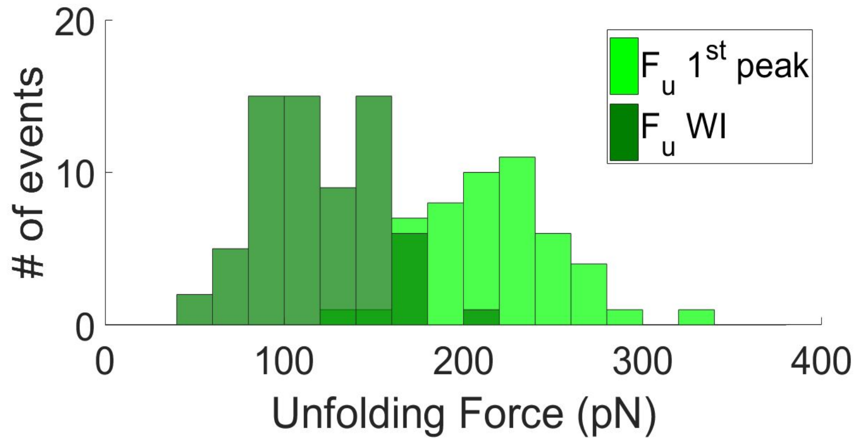

Figure S4: *Statistical distribution of the unfolding force F_U of the mechanically weak interactions compared to the unfolding force F_U of the corresponding first peak of the same force-extension curve (cumulative data for no ligand, DA and EGCG).*

Table S4 and Figure S5 illustrate the statistical distribution of F_U and L_C values for the mechanically weak interactions (WI), as obtained under the different tested conditions (no ligand, DA, ECGC). The data indicate that there is no appreciable effect of the ligands on WI F_U and L_C. As a consequence, the statistical values of F_U for WI are collectively evaluated for all the curves classified as WI (Fig.1D of the main paper).

	**No Ligands**	**200 μM DA**	**25 μM EGCG**	**Total**
**L_C (WI) - nm**	43 ± 19	41 ± 11	39 ± 12	41 ± 14
**F_U (WI) - pN**	111 ± 34	108 ± 37	136 ± 19	117 ± 34 (*)

Table S4: *Measured average values +/- standard deviation of the unfolding force F_U and of the contour length L_C measured for weak interaction peaks for each force-extension curve. The last column reports the cumulative statistical values from data acquired under the different tested conditions (no ligand, DA, EGCG) (* Fig.1D of the main paper).*

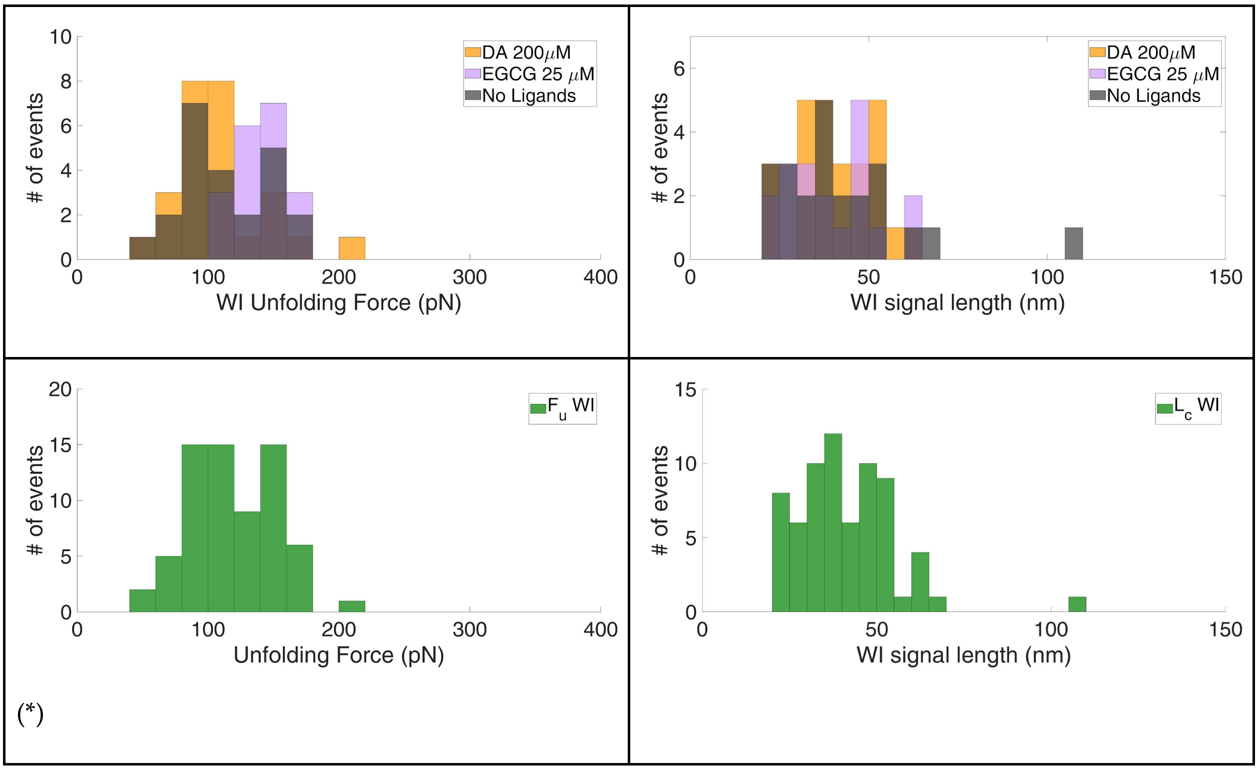

Figure S5 *Statistical distribution of the unfolding force* F_U *(left column) and contour length* L_C *(right column) measured for the WI peaks for each force-extension curve classified as WI. The upper panels represent results as a function of the different tested conditions (no ligand, DA, EGCG), as indicated by the color legend. The lower panels show the cumulative statistical distribution of the same data. (* Fig.1D of the main paper).*

Fig. S6 shows a scatter plot of the unfolding force F_U for all the WI peaks and their extension L_C. Data are shown as a function of the different tested conditions (no ligand, DA, ECGC). The curves characterized by weak interactions present up to three peaks. Data in Fig. S6 take into account one (circles), two (squares) or three (diamonds) peaks in each force-extension curve to build the scatter plot. The graph shows no correlation between the two parameters.

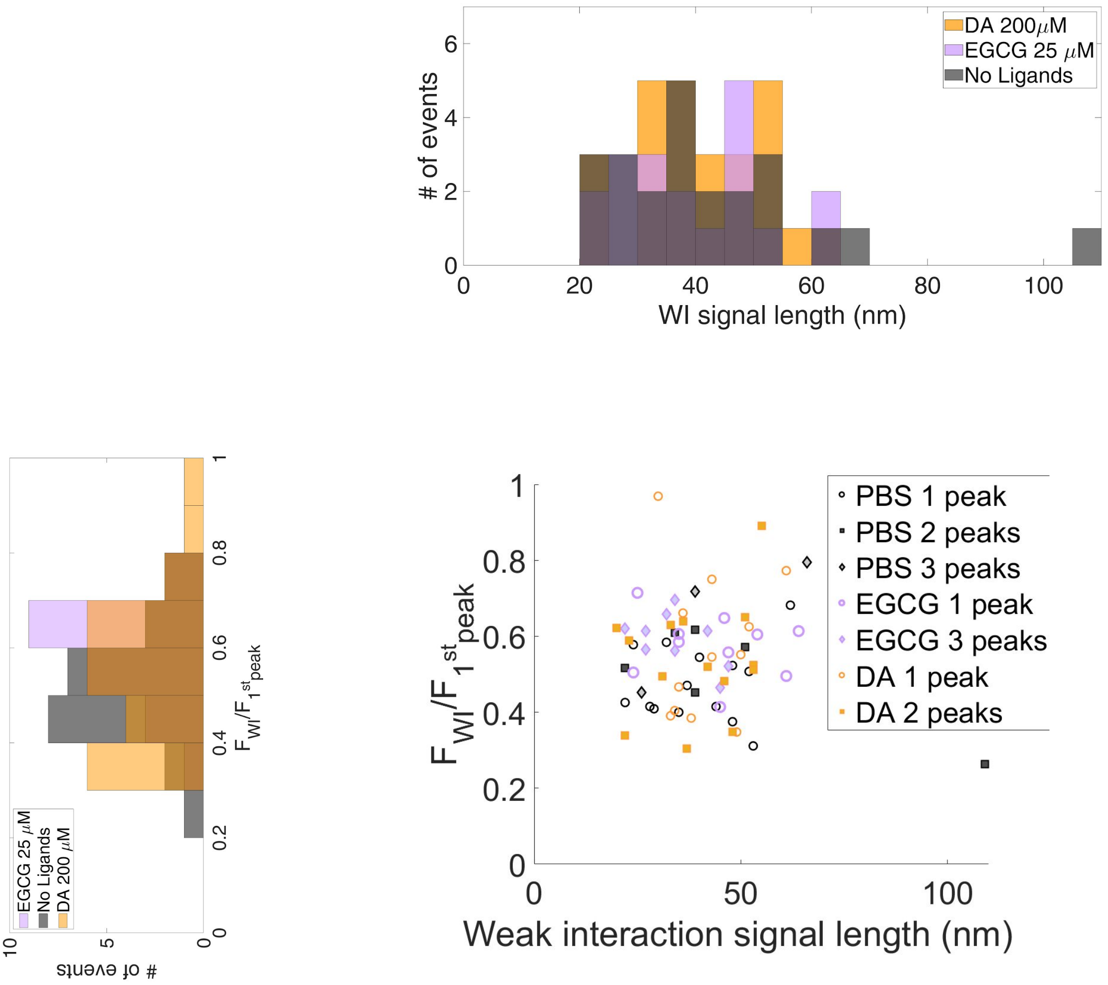

Figure S6: *Histograms and scatter plot of the unfolding force of the WI peaks and their corresponding contour length as a function of the different tested conditions (no ligand, DA, EGCG), as indicated by the color legend. Each dot of the scatter plot represents a single WI unfolding peak. Data take into account the number of unfolding peaks in each curve: a curve with a single peak contributes with one circle, a curve with two peaks contributes with two squares, a curve with three peaks contributes with three diamonds.*

Number of curves used for analysis of the conformational ensemble

	Number of curves		
	**No Ligands**	**200 μM DA**	**25 μM EGCG**
**Random Coil**	37	31	19
**Strong Interactions**	5	23	21
**Weak Interactions**	18	19	13
**N(tot)**	**60**	**73**	**53**

Table S5: *Number of curves used for data reported in Fig.2 of the main paper.*

	Percentage values (%)		
	**No Ligands**	**200 μM DA**	**25 μM EGCG**
**Random Coil**	62	42	36
**Strong Interactions**	8	32	40
**Weak Interactions**	30	26	24

Table S6: *Percentage values for SMFS data reported in Fig.2 of the main paper.*

Oxidative level of $(I27)_4$_α-syn_$(I27)_4$ polyprotein by mass spectrometry

To rule out that the conformational differences of $(I27)_4$_α-syn_$(I27)_4$ in the presence of DA or EGCG might be promoted by ligand-induced oxidation rather than ligand binding, the oxidative level of the polyprotein has been analyzed by MS-based proteomics techniques. $(I27)_4$_α-syn_$(I27)_4$ was incubated 4 hours in the absence of ligands and in the presence of either 200 µM DA or 25 µM ECGC, in order to mimic the conditions of SMFS experiments. Then, a fast digestion of the polyprotein was performed by adding trypsin (Sigma-Aldrich, St. Louis, MO) at protease:substrate 1:50 and incubating 30 minutes at 37 °C. The reaction was stopped by the addition of 1% formic acid, and the tryptic peptides were desalted by C18 Ziptip (Millipore, Burlington, MA) before injection into an Orbitrap Fusion mass spectrometer coupled to a nano-HPLC system (EASY-nLC 1000, Thermofisher, Waltham, MA). Peptides were separated on a C18 column (length 500 mm, ID 75 µm, particle size 2 µm) by a 90-minutes gradient, analyzed by MS/MS experiments and identified by the software Proteome Discoverer (Thermofisher), using methionine oxidation as a variable modification.

The sequence coverage was at least 92% for each sample run. The I27 domain contains one methionine residue (Met67). Wild-type AS contains four methionine residues (Met1, Met5, Met116 and Met127). However, Met1 has been removed in the construct employed in this work and the C-terminal ones, at positions 116 and 127, are known to be missed in a too large tryptic fragment under these conditions [43]. Thus, I27 Met67 and AS Met5 were used to monitor the oxidation level of the polyprotein. It has been shown that the oxidation kinetics of all AS Met residues is quite similar [43]. The extent of oxidation was quantified by the number of peptide-spectrum matches (PSMs) relative to the total PSMs for the oxidized and non-oxidized variants of each peptide. Both methionine residues feature low oxidation levels after the incubation (below 10% for AS Met5), and no induction by the ligands (Figure S7). This is in line with the evidence that DA- or EGCG-induced AS oxidation under similar conditions takes place on the time scale of days [43]. Thus, these results confirm that the effects of DA and EGCG revealed in this work by SMFS are ascribable to ligand binding DA or EGCG.

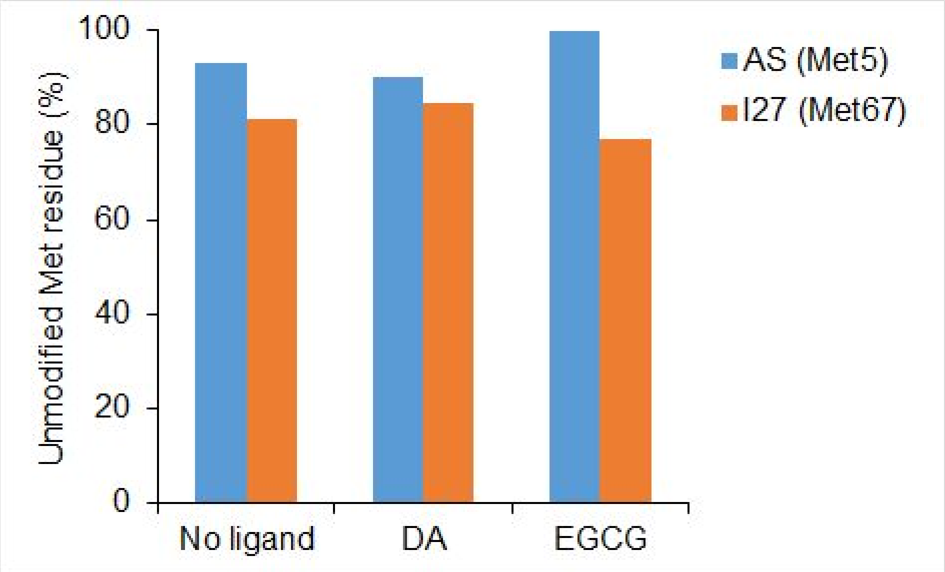

***Figure S7**: Percentage of non-oxidized methionine residues in the AS or I27 modules after 4h-incubation of $(I27)_4$_α-syn_$(I27)_4$ polyprotein in the absence of ligands or in the presence of DA or EGCG*

Supplementary Materials Bibliography

[43] E. Ponzini, A. De Palma, L. Cerboni, A. Natalello, R. Rossi, R. Moons, A. Konijnenberg, J. Narkiewicz, G. Legname, F. Sobott, P. Mauri, C. Santambrogio, R. Grandori, Methionine oxidation in α-synuclein inhibits its propensity for ordered secondary structure, J. Biol. Chem., 294 (2019) 5657-5665. https://doi.org/10.1074/jbc.ra118.001907.

[59] P.E. Marszalek, H. Lu, H. Li, M. Carrion-Vazquez, A.F. Oberhauser, K. Schulten, J.M. Fernandez, Mechanical unfolding intermediates in titin modules, Nature, 402 (1999), 100–103. https://doi.org/10.1038/47083.
